# Increased expression of Six1 correlates with progression and prognosis of prostate cancer

**DOI:** 10.1186/s12935-015-0215-z

**Published:** 2015-06-20

**Authors:** Jun Zeng, Rong Shi, Cui-xia Cai, Xin-rui Liu, Yan-bin Song, Min Wei, Wen-li Ma

**Affiliations:** Institute of Genetic Engineering, Southern Medical University, No.1838, Baiyun Road North, Guangzhou, People’s Republic of China

**Keywords:** Six1, IHC, Prostate cancer, Prognosis

## Abstract

Sineoculis homeobox homolog 1 (Six1), normally a developmentally restricted transcriptional regulator, is frequently dysregulated in mutiple cancers. Increasing evidences show that overexpression of Six1 plays a key role in tumorigenesis. However, the Six1 expression status and its relationship with the clinicopathological characteristics in prostate cancer were unclear. In this study, the mRNA and protein levels of Six1 in prostate cancer tissues and normal prostate tissues were evaluated. The clinicopathological significance of Six1 was investigated by immunohistochemistry (IHC) on a prostate cancer tissue microarray. The cut-off score for high expression of Six1 was determined by the receiver-operating characteristic (ROC) analysis. The correlation between Six1 protein expression and clinicopathological characteristics of prostate cancer was analyzed by Chi-square test. Increased expression of Six1 protein was observed in the majority of prostate cancer, compared with their paired adjacent normal prostate tissues. When Six1 high expression percentage was determined to be above 55 % (area under ROC curve = 0.881, *P* = 0.000), high expression of Six1 was observed in 55.6 % (80/144) of prostate cancer tissues and low expression of Six1 was observed in all normal prostate tissues by IHC. Increased expression of Six1 in patients was correlated with high histological grade (*χ*2 = 58.651, *P* = 0.00), advanced clinical stage (*χ*2 = 57.330, *P* = 0.000), high Gleason score (*χ*2 = 63.480, *P* = 0.000), high primary tumor grade (*χ*2 = 57.330, *P* = 0.000) and positive regional lymph node metastasis (*χ*2 = 19.294, *P* = 0.000). Furthermore, univariate and multivariate survival analysis suggested that Six1 was an independent prognostic indicator for overall survival (*P* < 0.05). This study suggests that Six1 could be served as an additional biomarker in identifying prostate cancer patients at risk of tumor progression, might potentially be used for predicting survival outcome of patients with prostate cancer.

## Introduction

Current diagnosis for prostate cancer includes digital rectal examination (DRE), prostate-specific antigen (PSA) and needle biopsy [1]. The appearance of prostate-specific antigen (PSA) testing has revolutionarily improved early prostate cancer detection. If rising PSA levels are detected, a needle biopsy of the prostate is recommended to inspect for histologic evidence of prostate cancer. If cancer is inspected, the patient can choose either active surveillance or one of several given treatment options, such as surgery, radiotherapy, or brachytherapy. However, current reports have raised concern over the efficacy of PSA testing. The U.S. prostate cancer screening trial report found that the mortality caused by prostate cancer was not reduced by PSA screening [2]. These studies suggested that PSA testing cannot distinguish between aggressive and indolent prostate cancer; therefore, many patients accepted overtreatment. As a result of these kind reports, the U.S. Preventive Services Task Force recommended to stop routine screening by PSA testing on all males [3]. Regardless of how much controversy about the PSA screening, there is an agreement that a definitive test is needed to distinguish patients with aggressive prostate cancer from the patients that have latent or indolent prostate cancer. Therefore, a critical question in the clinical activity of prostate cancer is how to distinguish the patients with indolent prostate cancer from patients with highly aggressive prostate cancer who would benefit from definitive treatment.

Homeobox genes encode transcription factors that are essential for the development of numerous organs and control processes such as proliferation, apoptosis, migration, and invasion [4–7]. The Six1 homeoprotein, a member of the Six family of homeodomain transcription factors, has been found overexpressed in mutiple cancers, including breast [8–10], rhabdomyosarcomas [11–13], hepatocellular carcinomas [14], ovarian [15] and Wilms tumors [16]. Recent evidence demonstrates that Six1 plays a role in cellular migration and invasion during embryogenesis [4, 17–20] and in breast cancer [21, 22] through a mechanism that may involve an EMT, which was associated with increased TGF-β receptor type I (TβRI) expression and Smad-dependent transforming growth factor-beta (TGF-β) signaling. In addition, Six1 overexpression enhances lymph node metastasis by increasing VEGF-C depending on TGF-β signaling in cervical cancer [23–26]. Forementioned studies suggested that Six1 enhances cancer cell proliferation and shortens survival and its overexpression in immortalized mammary epithelial cells induces EMT, leading to highly aggressive and invasive tumors when transplanted into nude mice [7].

Noteworthy, HD Jin *et al.* reported that Six1 overexpression positively correlated with clinical stage, lymph node metastasis, overall survival (OS) and disease-free survival (DFS) rates of patients with breast cancer. Moreover, patients with high Six1 expression had poorer prognosis than those with low Six1 expression in late stage breast cancer cases [27]. However, the relationship between Six1 and the clinicopathological characteristics of prostate cancer has not previously been examined.

## Materials and methods

### Patient specimens and prostate cancer cohorts

A total of 144 paraffin-embedded tissues diagnosed from 2002 to 2012 were retrieved for tissue microarray (TMA) construction and immunohistochemistry analysis. All the samples were collected from the Department of Urology, Nanfang Hospital (Guangzhou, China). The samples selected were pathologically diagnosed with prostate cancer, having received no prior chemotherapy or radiotherapy before surgery. Ages of the 144 patients with prostate cancer varied from 44 to 77 years (median, 65 years), clinicopathological features of patients including age at diagnosis, histologic grade, clinical stage, gleason score and pTNM stages. Written informed consent was obtained from all patients for use of the tissue samples and clinical records. The study protocol was performed under the approval by the Ethic Committee of the Nanfang Hospital. All cases were evaluated by experienced pathologists for histopathological grading.

### Quantitative real-time PCR

qRT-PCR was performed to determine the expression of Six1 messenger RNA (mRNA). Briefly, we isolated total RNAs from frozen tissues using TRIzol Reagent according to the manufacturer’s protocol (Life Technologies) and reverse transcribed to generate cDNA (PrimeScript RT-PCR Kit, Takara Bio). β-Actin was used as an internal control. The levels of mRNA encoding were quantified by real-time PCR with the Applied Biosystems 7500 Fast Real-Time PCR System using SYBR Premix Ex Taq (Applied Takara Bio). The sequences of the primers were as follows: Six1 forward 5’- TTCTCGCCTCACAACCACCCCA-3’ and reverse 5’-TACCACTCCCGCAGGACACCCC-3’ and β-actin forward 5’-TGGCACCCAGCACAATGAA-3’ and reverse 5’-CTAAGTCATAGTCCGCCTAGAAGCA-3’. The PCR conditions were as following procedures: initial step 95 °C for 2 min, followed by 35 cycles of 95 °C for 30 s, 56 °C for 30 s, and 72 °C for 2 min and a final elongation step of 72 °C for 5 min. All qRT-PCRs were repeated three times. Relative quantification of Six1 mRNA expression was calculated using the 2^−ΔΔCT^ method.

### Western blotting analysis

Total proteins from 8 pairs of fresh prostate cancer and adjacent normal tissues were extracted by radio-immunoprecipitation assay (RIPA) buffer containing 1 mM phenylmethanesulfonyl fluoride (PMSF) (Beyotime, Haimen, China). After centrifugation, supernatant was collected and treated with BCA Protein Assay Kit (Beyotime, Haimen, China). The protein concentration was then measured at 562 nm -by the TECAN Infinite 200 microplate reader (TECAN, Switzerland). Tissue homogenates (35 μg protein for each sample) were separated by 10 % sodium dodecyl sulfate polyacrylamide gel electrophoresis (SDS-PAGE), and the resolved proteins were transferred onto a polyvinylidene difluoride (PVDF) membrane (Millipore, USA) by a Trans-Blot SD Semi-dry transfer cell machine (Bio-Rad, USA). After the blots were washed with 1 × TBST buffer (10 mM Tris-HCl [pH 7.6], 150 mM NaCl, and 0.05 % Tween-20), the membranes were blocked overnight with 5 % skim milk and incubated with the appropriate primary antibody at room temperature for 2 h. Polyclonal rabbit anti-human antibody against Six1 (Atlas antibody, Sweden, 1:500), and monoclonal rabbit anti-human antibody against β-actin (Cell Signaling Technology, Danvers, MA, USA, 1:3000) were used for detecting the protein level of Six1 and β-actin in each sample. The membranes were then washed by 1 × TBST, primary antibodies were detected with goat anti-rabbit IgG conjugated to horseradish peroxidase (HRP) (Santa Cruz Biotechnology, Cal, USA, 1:5000), and the bands were detected by BeyoECL Plus Kit (Beyotime, Haimen, China). Finally, the results of western blotting were visualized by the Image Station 4000R PRO scanner (CareStream Health, Rochester, NY, U.S.A.).

### Tissue microarray (TMA) construction and immunohistochemistry (IHC)

Representative sections of prostate cancer or normal prostate tissue in the pre-existing paraffin-embedded tissue blocks were determined according to the overlaid H&E staining slides. The TMA was constructed by using a needle to punch a 1.5 mm diameter cylinder in the representative section of each block, and placing the cylinders into an array on a recipient paraffin block. 5 μm thick multiple sections were cut from the TMA block and mounted on microscope slides for immuohistochemistry analysis. The TMA consisted of a total of 144 cases of prostate cancer and 10 cases of normal control paraffin-embedded tissue. Clinical characteristic about the patients was summarized in Table 1. The TMA slide was dried overnight at 37 °C, deparaffinized in xylene, rehydrated through graded alcohol, and then immersed in 3 % hydrogen peroxide for 20 min to block the endogenous peroxidase activity, and antigen-retrieved by microwave heating with sodium citrate buffer (pH = 6.0) at 100 °C for 30 min. Then the slides were pre-incubated with 10 % normal goat serum at room temperature for 30 min to reduce nonspecific reaction. The primary rabbit anti-Six1 polyclonal antibody (Atlas antibody, Sweden) was diluted (1:1000) with 1 × phosphate buffered saline (PBS) and applied overnight in a humidity chamber at 4 °C. The slide was sequentially incubated with a polymer peroxidaselabeled secondary antibody (ZSGB-Bio, Beijing, China) for 30 min at room temperature, and then stained with DAB Horseradish Peroxidase Color Development Kit (Beyotime, Haimen, China). Finally, the sections were counterstained by hematoxylin. Known IHC positive slide was used as a positive control, and phosphate buffered saline replaced anti-Six1 primary antibody was used as a control.

**Table 1:** Association of Six1 expression with patients’ clinicopathologic features in prostate cancer

		Six1 protein			
Variable	All Cases	Low	High	*χ*2	*P* value^a^
Age^b^					
≤65	68	31	37	0.794	0.068
>65	76	33	43		
Histological grade					
G1 + G2	77	57	20	58.651	0.000
G3	67	7	60		
Clinical stage					
I + II	40	38	2	57.330	0.000
III + IV	104	26	78		
Gleason score					
1–7	54	47	7	63.480	0.000
8–10	90	17	73		
pT status					
T1 + T2	40	38	2	57.330	0.000
T3 + T4	104	26	78		
pN status					
N0	107	59	48	19.294	0.000
N1 + N2	37	5	32		
pM status					
M0	105	48	57	0.253	0.615
M1	39	16	23		

^a^Chi-squared test

^b^Mean age

### Evaluation of IHC

Immunoreactivity for the Six1 protein was scored by a semi-quantitative method by recording the proportion of positive tumor cells over the total number of tumor cells. Scores were assigned by using 5 % increments (0, 5…100 %). The reproducibility of the scoring method between pathologists has been described previously for TMAs [28–31]. The scores were accepted if two of the three investigators (M.W, R.S and J.Z) agree with the values. Otherwise, the values were re-estimated until a consensus was reached. Our conclusions were in complete agreement in 80 % of the cases, which indicated that the scoring method was highly reproducible.

### Selection of Cut-off scores using receiver- operating characteristic (ROC) curves

Receiver operating characteristic curve analysis was utilized to determine the cut-off score by using the 0, 1-criterion [32]. At the Six1 score, the sensitivity and specificity for each outcome under study was plotted, thus generating various ROC curves. The score was selected as the cut-off score, which was closest to the point with both maximum sensitivity and specificity. Tumors designated as “low” for Six1 were those with scores below and equal to the cut-off value, whereas “high” tumors were those with scores above the value [29]. In order to perform ROC curve analysis, the clinicopathological features were dichotomized: age (≤65 or >65), histological grade (low [G_1_ + G2] or high G_3_), clinical stage ([I + II] or above [III + IV]), Gleason stage (1–7 or 8–10), T stage (early [T_1_ + T_2_] or late [T_3_ + T_4_]), N stage (N_0_ [no lymph node involvement] or N_1_ + N_2_ [any lymph node involvement]), M stage (M_0_ [no distant metastasis] or M_1_ [distant metastasis]).

### Statistical analysis

Statistical analysis was performed by using the SPSS statistical software program (standard version 18.0; SPSS, Chicago, USA). The relationship between Six1 protein expression and clinicopathological data of Prostate cancer patients was estimated using the *χ*^2^ test. ROC curve analysis was applied to determine the cut-off score for Six1 positivity, and the areas under curves (AUCs) were then calculated. The association between survival and each variable was determined with the Kaplan-Meier method (the log-rank test). Multiple Cox proportional hazards regression was carried out to identify the independent prognostic factors. *P* < 0.05 (two-tailed) denotes the presence of a statistically significant difference.

## Results

### The expression level of Six1 in prostate cancer and adjacent normal prostate tissues detected by western blotting and IHC

**Fig. 1:**
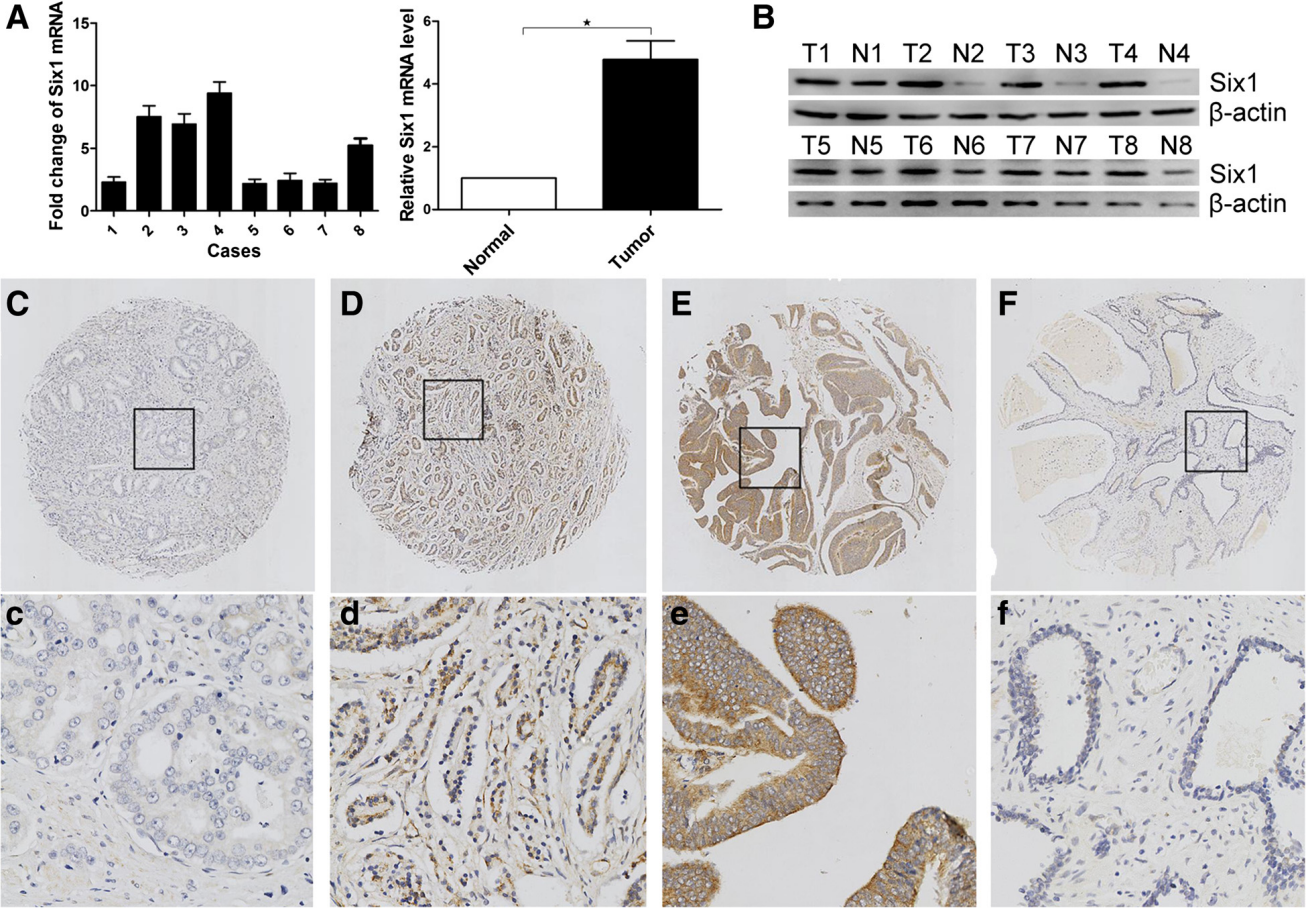
Expression of Six1 in prostate cancer tissues and normal prostate tissues detected by western blotting and immunochemistry. **a** Relative mRNA expression of Six1 normalized to β-actin was calculated (*n* = 8), ^★^ indicates *P* < 0.05. **b** Expressions of Six1 protein in 8 paired tissues were examined by western blotting. Micrographs showed weak (**c, c**), moderate (**d, d**), and strong (**e, e**) staining of Six1 in prostate cancer tissues, as well as low (**f, f**) expression of Six1 in normal prostate tissues (upper magnification × 100, lower panel: magnification × 400.)

In this study, the protein and/or mRNA expression of Six1 was first examined by Western blotting and/or qRT-PCR in 8 pairs of primary prostate cancer and adjacent normal prostate tissues. A significantly increase in both protein and mRNA expression of Six1 was detected in prostate cancer tissues compared to adjacent nontumorous tissues (Fig. 1a and b). For Six1 IHC staining in prostate cancer tissues and normal prostate tissues, immunoreactivity was seen primarily in the cytoplasm and perinucleus of prostate cancer cells (Fig. 1). Six1 expression could be evaluated informatively in 144 prostate cancer cases by the TMA constructed previously and in 10 normal prostates. Immunoreactivity ranged from 0 to 100 %. According to ROC analysis, expression percentage for Six1 above the critical value 55 % was defined as high expression. The high expression of Six1 was detected in 80 ⁄ 144 (55.6 %) of prostate cancer cases. The decreasing frequency of Six1 high expression was detected in normal prostate tissues (Fig. 1).

### Selection of Six1 cut-off scores

**Fig. 2:**
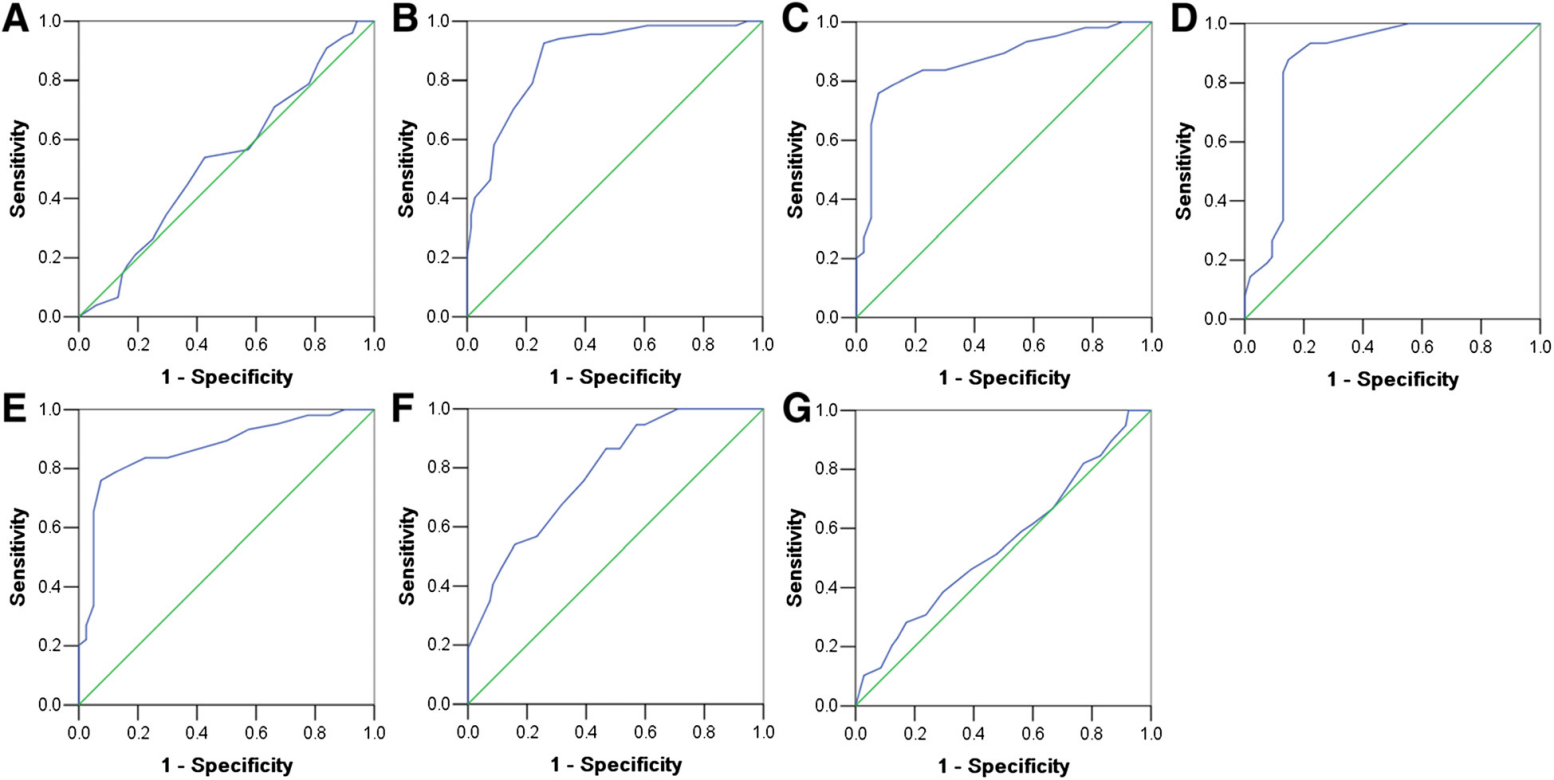
Determination of the cutoff value of high Six1 expression in prostate cancer tissues by ROC curves. The sensitivity and 1-specificity were plotted for each clinical characteristic, including age (**a**), pathological grade (**b**), clinical stage (**c**), Gleason grade (**d**), pT status (**e**), pN status (**f**), pM status (**g**)

**Fig. 3:**
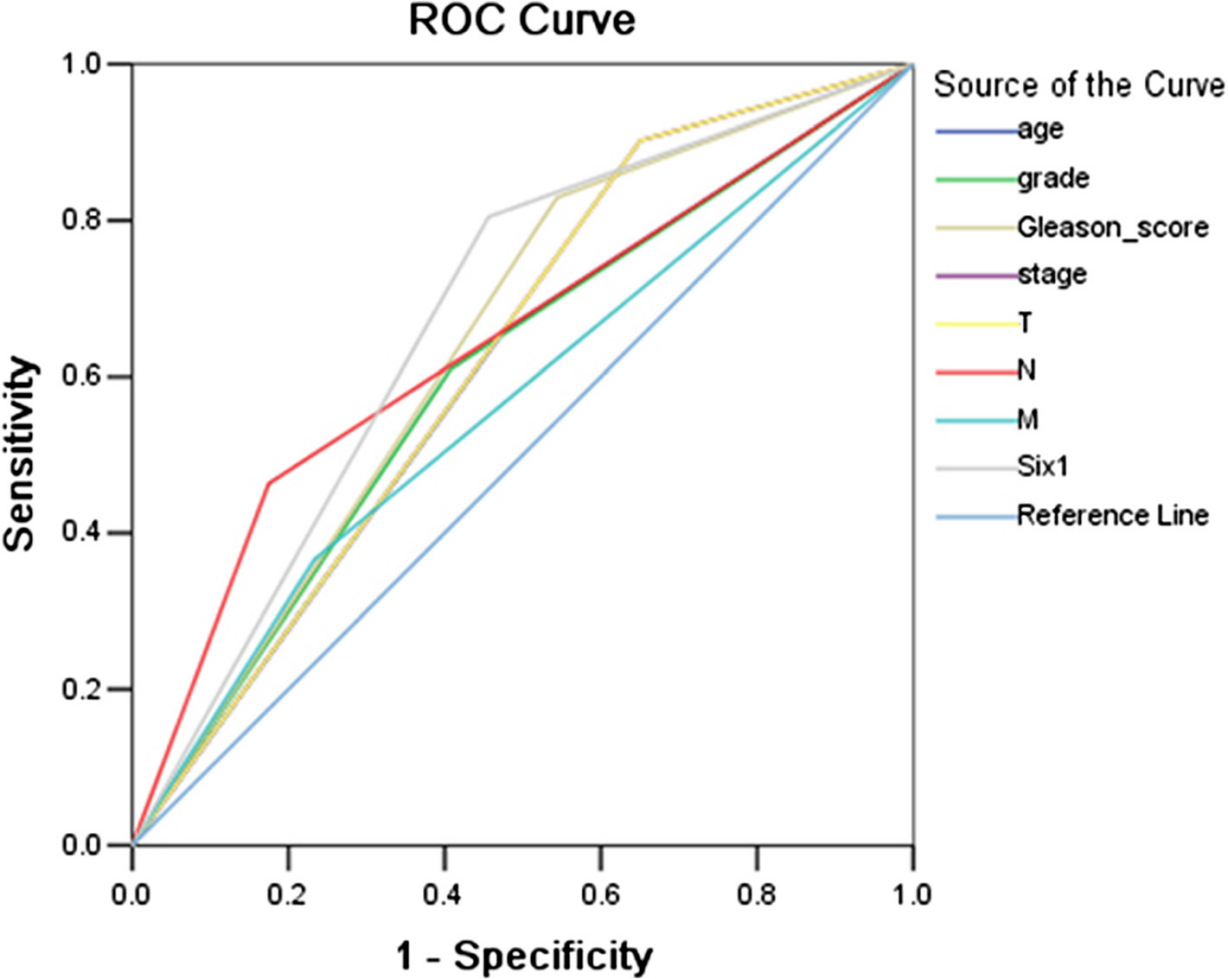
Receiver–operator curve (ROC) analysis for different clinicopathological parameters and Six1 expression was used to evaluate the survival status. clinical stage (AUC = 0.626, *P* = 0.019), Gleason_score (AUC = 0.643, *P* = 0.008), T stage (AUC = 0.626, *P* = 0.019), N stage (AUC = 0.644, *P* = 0.007), Six1 expression (AUC = 0.674, *P* = 0.001) implied significant statistical associations with the survival

The ROC for each clinicopathological parameter (Fig. 2) clearly show the point on the curve closest to (0.0, 1.0) which maximizes both sensitivity and specificity for the outcome. The analysis of ROC for each clinicopathological feature and Six1 expression (AUC = 0.674, *P* = 0.001) is carried out to evaluate the patients’ survival status (Fig. 3). The cut-off score was determined to be above 55 % for Six1 high expression. Tumor tissues with scores above the determined cut-off values were considered high expression of Six1 protein leading to the greatest number of tumor cases correctly classified as having or not having the clinical outcome. The corresponding AUCs (95 % confidence interval [CI]) are showed in Table 2.

**Table 2:** Area under the ROC curve values for each clinicopathological feature

Feature	AUC (95 % CI)	*p* value
Age	0.531	0.518
Histological grade	0.881	0.000
Clinical stage	0.869	0.000
Gleason score	0.874	0.000
pT	0.869	0.000
pN	0.784	0.000
pM	0.505	0.373

*AUC*, Area under the curve; *CI*, Confidence interval

### Correlations between Six1 protein expression and clinicopathological parameters of prostate cancer

Six1 protein overexpression was significantly correlated with histological grade, clinical stage, Gleason score, primary tumor statues and regional lymph node metastasis of prostate cancer (*P* < 0.001). However, Six1 protein expression was not related with patient age, distant metastasis in prostate cancer (*P* > 0.05) (Table 1). For histological grades, the high expression rate of Six1 protein was significantly higher in late grade (G3) prostate cancers (89.6 %, 60/67) than in early histological grade cases (G1 + G2) (26.0 %, 20/77) (*P* < 0.001). Similarly, the high expression rate of Six1 protein was higher in breast cancers with high Clinical stage (75.0 %, 78/104) compared with those with low Clinical stage (0.5 %, 2/40) (*P* < 0.001). The high expression rate of Six1 protein was significantly higher in high Gleason score (8–10) prostate cancers (81.1 %, 73/90) than in low Gleason score cases (1–7) (13.0 %, 7/54) (*P* < 0.001). The high expression rate of Six1 protein was 75.0 % (78/104) in high pT grade, which was significantly higher than in low pT grade (0.5 %, 2/40) (*P* < 0.001). Meanwhile, the high expression rate of Six1 protein was significantly higher in high pN grade (N1 + N2) prostate cancers (81.1 %, 73/90) than in low pN grade cases (N0) (13.0 %, 7/54) (*P* < 0.001).

### Relationship between clinicopathologic variables, Six1 protein expression and prostate patients’ survival

**Fig. 4:**
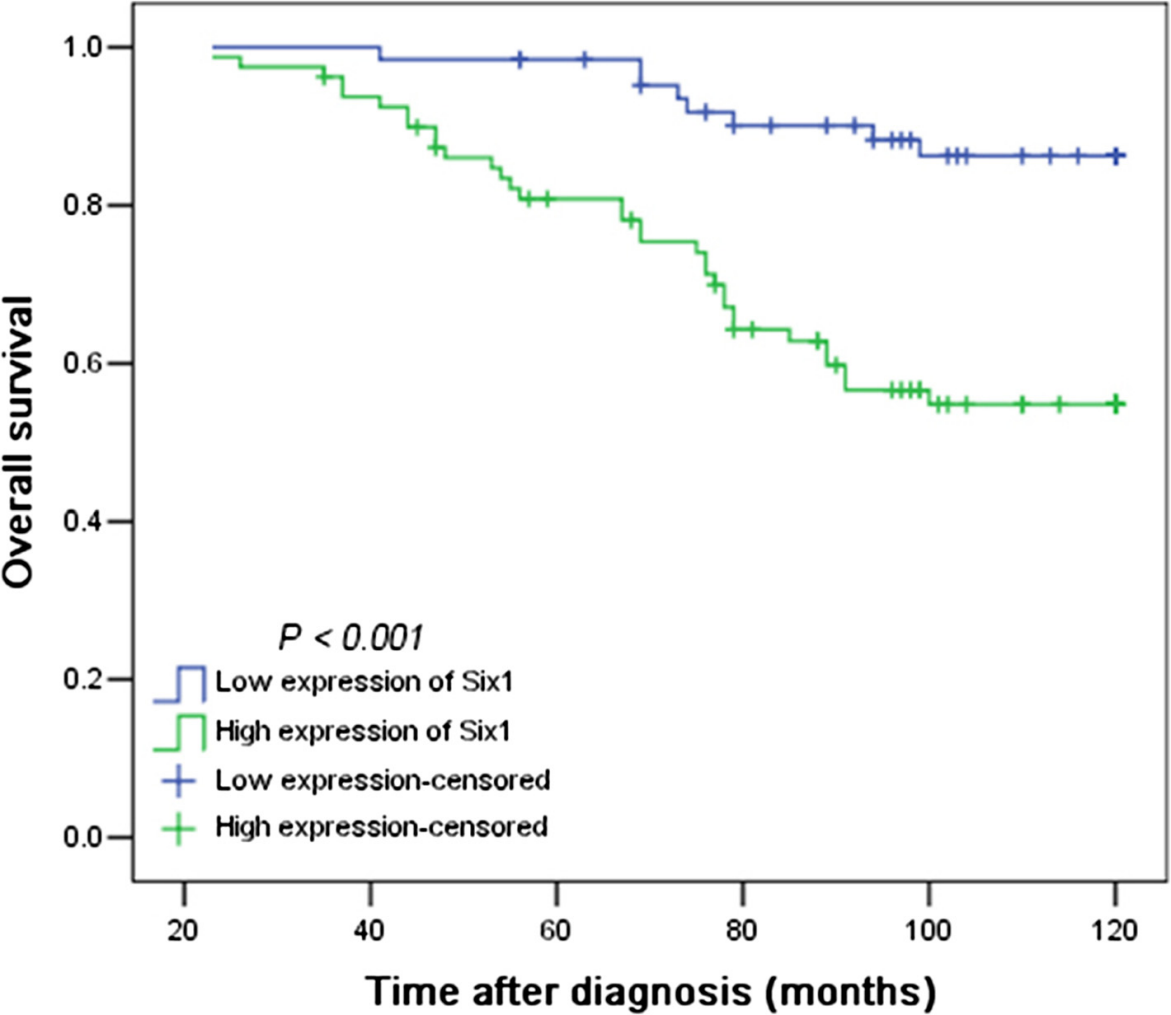
Kaplan–Meier survival analysis of Six1 expression in 144 patients of prostate cancer (log-rank test). Relationship of Six1 expression and patient overall survival: low expression, *n* = 64 and high expression, *n* = 80 (*P* < 0.001)

In univariate analysis, Kaplan–Meier survival curves and the *P*-values for these curves were determined by log-rank method. Above all, to confirm the representativeness of the prostate cancer in the study, we analyzed established prognostic factors of patient survival. The univariate analysis demonstrated a significant impact of well-known clinicopathological prognostic parameters, such as histolotical grade, clinical stage, Gleason score, pT status, pN status and Six1 expression on patient overall survival (Table 3). Assessment of survival in all specimens demonstrated that increased expression of Six1 protein was associated with worse overall survival (*P <* 0.001, Fig. 4), and the mean survival time for patients with tumors having low Six1 expression was 87.4 months compared to 42.8 months for patients with tumors having high Six1 expression (Table 3).

**Table 3:** Univariate and Multivariate Analysis of Different Prognostic Features in 144 Patients with prostate cancer

		Univariate analysis^a^			Multivariate analysis^b^	
Variable	All case	Mean (months)	*χ* ^2^	*P* value	HR (95 % CI)	*P* value
Age^c^			6.487	0.011	2.220 (1.146–4.300)	0.018
≤65	68	111.567				
>65	76	96.475				
Histological grade			4.312	0.038	0.696 (0.335–1.446)	0.331
G1 + G2	77	106.956				
G3	67	99.493				
Clinical stage			9.381	0.002	1.369 (0.312–6.004)	0.677
I + II	40	114.868				
III + IV	104	99.086				
Gleason score			9.273	0.002	1.246 (0.391–3.969)	0.710
1–7	54	111.779				
8–10	90	98.610				
pT status			9.381	0.002	1.369 (0.312–6.004)	0.677
T1 + T2	40	114.868				
T3 + T4	104	99.086				
pN status			13.772	0.000	1.710 (0.803–3.642)	0.164
N0	107	107.933				
N1 + N2	37	90.843				
pM status			1.529	0.216	1.162 (0.554–2.437)	0.692
M0	107	104.893				
M1	37	100.275				
Six1 expression			16.051	0.000	3.434 (1.168–10.100)	0.025
Low	64	113.943				
High	80	94.992				

*HR* indicates hazards ratio; *CI* indicates confidence interval

^a^Log-rank test; ^b^Cox regression model; ^c^Mean age

### Independent prognostic factors of prostate cancer: multivariate survival analysis

A multivariate analysis based on the Cox proportional hazard model was applied to test the independent value of each parameter predicting overall survival (Table 3). Expressions of Six1 as well as other clinicopathological features that were significant by univariate analysis (histological grade, clinical stage, Gleason score, pT stage and pN stage) were included in multivariate analysis. The expression of Six1 was found to be an independent prognostic factor for adverse overall survival (relative risk, 2.322; 95 % CI, 1.163-4.636; *P* = 0.017). Of the other parameters, Clinical stage (*P* = 0.044), pT stage (*P* = 0.044) were demonstrated as well an independent prognostic factor for overall survival.

## Discussion

Prostate cancer is one of the most frequently diagnosed malignancies and second only to lung and bronchus cancer in men worldwide. Approximately 258,400 men dead from this disease in 2008 [33]. At current rates of diagnosis, one-sixth of men will be diagnosed with prostate cancer during his lifetime [34]. From the clinical perspective, the tumors in different patients must be varying in the molecular level and the aim of personalized medicine is to generate individual risk profiles from the primary prostate cancer that could distinguish high-risk individuals who should accept aggressive therapeutic treatment and clinical follow-up from those who have indolent prostate cancer to avoid these individuals from undergoing overtreatment. Recently, several studies have investigated molecular and genetic characteristics of prostate cancer to develop both predictive and prognostic biomarkers [35–37].

It was reported that in multiple types of human cancer, such as breast, rhabdomyosarcomas, hepatocellular carcinomas, ovarian, and Wilms tumors, overexpression of Six1 was frequently identified [8–16]. In the study, we investigated the expression patterns of Six1, by Western blotting using fresh prostate tissues, and by IHC using TMA containing a large cohort of prostate tumor samples (144 cases) with complete clinicopathological and follow-up data. Western blotting revealed that up-regulation expression of Six1 was detected in prostate cancer, when compared with adjacent normal prostate tissues. Moreover, the IHC results demonstrated that an increasing expression of Six1 was observed from low malignant tumors to high malignant tumors. Six1 immunoreactivity was assessed using a scoring method described in previous study [29]. The reliability of this scoring method for Six1 was assessed by three investigators and was again found to be highly reproducible. To select the IHC cut-off scores for Six1 positivity, ROC analysis was carried out for each of the clinicopathological parameters, including age, histological grade, clinical stage, Gleason score, pTNM stage. ROC analysis for different clinicopathological features and Six1 expression was also used to evaluate the survival status. Six1 expression demonstrated significant statistical associations with the survival status of patients with prostate cancer. These data provided evidence that the unregulated expression of Six1 played an important role in tumorigenic process of multiple human cancers, including prostate cancer.

In summary, the study suggests that increased expression of Six1 may represent a more aggressive status of prostate cancer. In addition, these results show that increased expression of Six1 predicts poorer outcome of the cancer for the patients compared to those patients with decreased expression of Six1. Further studies are needed to clarify the precise mechanism of Six1 in prostate cancer and to develop potential therapies targeting Six1 in prostate cancer.
